# Progress in Disease-Modifying Therapies for Parkinson's Disease

**DOI:** 10.14336/AD.2025.0627

**Published:** 2026-09-04

**Authors:** Shuyuan Zhang, Gang Shao, Bin Wu, Liang Xia, Lei Wang, Liwen Li, Kai Jin, Yangfan Zou, Caixing Sun

**Affiliations:** ^1^Zhejiang Cancer Hospital, Hangzhou, Zhejiang 310022, China.; ^2^Hangzhou Institute of Medicine (HIM), Chinese Academy of Sciences, Hangzhou, Zhejiang 310018, China.; ^3^Zhejiang Provincial Key Laboratory of Silkworm Bioreactor and Biomedicine, College of Life Sciences and Medicine, Zhejiang Sci-Tech University, Hangzhou, 310018, China

**Keywords:** Parkinson's disease, Disease-modifying therapies, Therapeutic targets, Research progress

## Abstract

Parkinson's disease (PD) is a highly prevalent neurodegenerative disorder, and current therapeutic approaches fail to prevent the progressive loss of dopaminergic neurons. To date, no disease-modifying therapies (DMTs) have been approved for PD. Developing effective DMTs remains the foremost objective in PD research. Here, we review the rationale for α-synuclein, LRRK2, GBA1, the PINK1-Parkin axis, and GLP-1R as potential therapeutic targets for PD. Additionally, we summarize the functional alterations observed in cellular organelles, including mitochondria, lysosomes, and the endoplasmic reticulum, in the context of PD. We also highlight the progress in drug development targeting these therapeutic candidates and the associated organelles. Furthermore, we discuss the advancements in stem cell-based therapeutic strategies in the field of PD research. It is believed that deepening understanding of disease mechanisms, combined with the development of novel technologies, offers promising potential for more effective solutions to this debilitating condition.

## 1. Introduction

Parkinson's disease (PD) is the second most common neurodegenerative disorder worldwide. Recent studies indicate that the global prevalence of PD is 1.51 per 1,000 people, affecting approximately 10 million individuals [1]. Age is the most significant risk factor for PD [2], with approximately 1.7% of the global population aged 65 and above being affected, and around 4-5% of people over 85 years of age. The incidence of PD in men is 1.5 times higher than in women [3]. Other risk factors include environmental exposure, diet, trauma, and metabolic factors. Studies analyzing both monozygotic and dizygotic twins have shown a heritability of 27% for PD [4]. Since the discovery of the SNCA gene mutation in 1997 [5], more than 100 genes or genetic loci have been identified [6-8].

## 2. Current Treatment of Parkinson's Disease

The hallmarks of PD pathology include the degeneration of dopaminergic neurons in the substantia nigra pars compacta (SNc) [9] and the intracellular deposits of α-synuclein, leading to the formation of Lewy bodies [10]. PD is characterized by both motor and non-motor symptoms. Motor symptoms primarily include bradykinesia, rigidity, resting tremors, and postural instability, all of which severely impair the life quality of patients [11]. Non-motor symptoms, such as neuropsychiatric disturbances, hyposmia, sleep disorders, autonomic dysfunction, and cognitive decline [12], usually appear ahead of motor symptoms and are of great importance for early diagnosis [13]. The diagnosis and evaluation of PD mainly bases on clinical history taking, physical examination [12-14] and questionnaires including the Movement Disorder Society Unified Parkinson's Disease Rating Scale (MDS-UPDRS) [15]. Dopamine transporter single-photon emission computed tomography (DAT-SPECT) can aid in the diagnosis [16], particularly in differentiating PD from other disorders like essential tremor [17]. In addition, skin biopsy is considered an effective method for the histopathological diagnosis of PD [18-20].

Current clinical treatment PD primarily relies on the symptomatic treatments for motor and non-motor symptoms. The treatments for motor symptoms include the use of levodopa and its combination formulations, monoamine oxidase B inhibitors, dopamine agonists, anticholinergic medications, and catechol-O-methyltransferase inhibitors [12]. Since the last century, deep brain stimulation (DBS) has emerged as another effective surgical intervention for managing motor disabilities and drug-resistant tremors in advanced PD patients [21]. Non-motor symptoms of PD affect multiple systems and may be more debilitating than motor symptoms. Some non-motor symptoms are caused by medications used to treat motor symptoms, requiring adjustments in dosage or treatment regimens. Other non-motor symptoms are managed with specialized pharmacological treatments tailored to the specific symptoms.

Symptomatic treatments cannot prevent further loss of dopaminergic neurons in PD. As the duration of medication increases, the therapeutic window gradually narrows, and side effects such as levodopa-induced dyskinesia (LID), excessive daytime sleepiness, orthostatic hypotension, and impulse control disorders become more prominent [22]. Therefore, the development of disease-modifying therapies (DMTs) has become a key objective, aiming to slow down or even reverse the neurodegenerative process, which represents a significant unmet medical need.

## 3. Progress in Disease-Modifying Therapies for Parkinson's Disease

**Figure 1.**
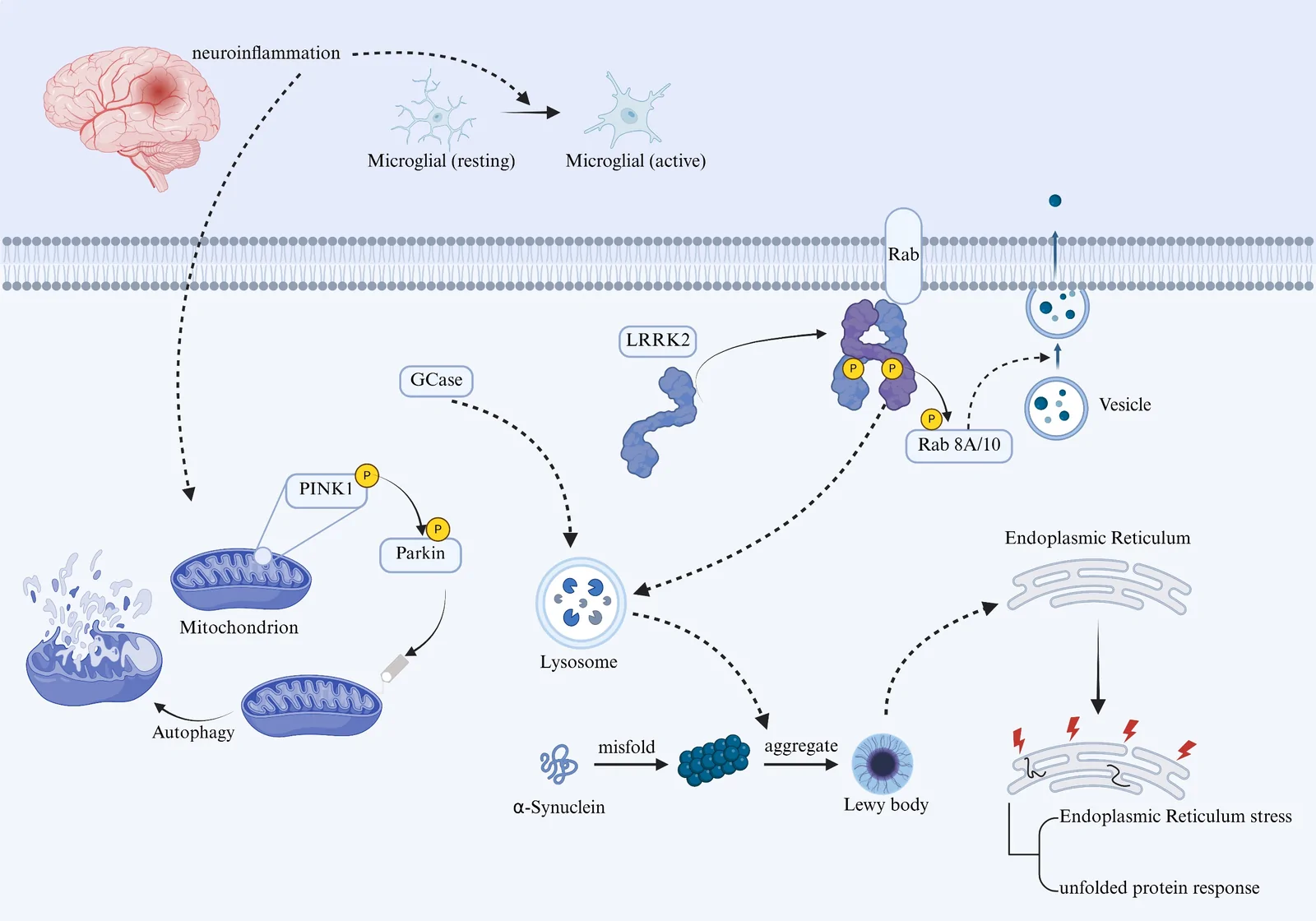
**The basis of DMT development for PD**. Created with BioRender.

DMTs refer to the interventions that target the underlying pathological mechanisms of a disease to alter its progression and outcomes, rather than merely alleviating symptoms. The feasibility of DMTs is based on an in-depth understanding of the disease mechanisms that is rapidly evolving. In merely 30 years, research into the pathological alterations of α-synuclein and its mechanisms of cellular damage, the identification of PD risk genes such as *SNCA*, *Leucine-Rich Repeat Kinase 2* (*LRRK2*), *Glucosylceramidase Beta 1* (*GBA1*), *PRKN*, and *PTEN-induced kinase 1* (*PINK1*), as well as the functional changes in cellular organelles such as mitochondria, lysosomes, and the endoplasmic reticulum in disease progression, form the basis for DMT development (Fig. 1).

In recent years, the development and application of new technologies have provided additional therapeutic strategies for DMTs. These include the development of novel animal models, monoclonal antibody therapies, antisense oligonucleotides (ASOs), small interfering RNAs (siRNAs), CRISPR/Cas9 gene editing, Proteolysis Targeting Chimeras (PROTACs), adeno-associated virus (AAV) vectors, and stem cell-based therapy. Similar to PD, Alzheimer's disease is a neurodegenerative condition with protein aggregation, and the promising results from Alzheimer's disease DMT research targeting β-amyloid protein offer valuable insights for the development of DMTs in the PD field. Some DMT strategies have entered clinical trials (Table 1).

### 3.1 α-Synuclein

In 1912, Fritz Heinrich Lewy first reported protein inclusions in the neuronal cytoplasm, which was soon named as "Lewy bodies" [23]. In 1997, α-synuclein was confirmed as the major component of Lewy bodies [24] and point mutations in the SNCA gene were first identified in familial PD[5]. α-Synuclein is a protein composed of only 140 amino acids. α-synuclein misfolding, particularly the phosphorylation at serine 129 [25, 26] or the protein mutation [27, 28], gradually aggregates to form the Lewy body in the brain. The precise mechanisms by which α-synuclein oligomers cause cell death are not fully understood, but they likely involve disrupted protein homeostasis, endoplasmic reticulum stress, and glutamate receptor dysfunction [29], *etc*. Aggregation of α-synuclein may also propagates between neurons via exosomes [30], further contributing to neurodegeneration. Therefore, targeting α-synuclein has become a critical direction for disease-modifying treatments in PD.

Among these therapeutic strategies, monoclonal antibody therapy has garnered the most attention. Typically, BIIB054 (Cinpanemab) [31] and PRX002 (Prasinezumab) [32] have been developed to prevent the aggregation of α-synuclein. The double-blind phase Ⅱ clinical trial of BIIB054 (PARK study) enrolled 357 early-stage PD patients. The patients were randomly assigned to receive either 250 mg, 1250 mg, or 3500 mg of Cinpanemab or a placebo, administered every 4 weeks for 52 weeks, followed by an extension phase up to 112 weeks. However, the trial was terminated after a mid-study analysis at 72 weeks revealed no improvement in MDS-UPDRS scores in the treatment group compared to placebo, indicating a lack of efficacy [33]. Cinpanemab recognizes the N-terminus and has low binding affinity to monomeric α-synuclein, such that this single therapy may not be sufficient to slow disease progression. A phase Ⅱ randomized, double-blind clinical trial that began in June 2017, aimed to evaluate the efficacy of PRX002 (PASADENA study). A total of 316 early-stage PD patients were enrolled. In the first phase, patients received 1500 mg or 4500 mg of Prasinezumab or a placebo for 52 weeks. The results showed no significant effect on MDS-UPDRS scores, or dopamine transporter levels as measured by SPECT [34]. However, in patients with rapid disease progression, a reduction in MDS-UPDRS Part III score was observed after one year of treatment [35]. The second phase involved randomizing the placebo group to receive 1500 mg or 4500 mg of Prasinezumab for 52 weeks. In the ongoing third phase, all participants are receiving 1500 mg of Prasinezumab for 5 years. Preliminary results indicate that patients treated with Prasinezumab showed slower motor progression at year 4. Notably, compared to the Parkinson’s Progression Markers Initiative (PPMI) external comparator, patients who received the drug from the start of the trial showed a 118% improvement in MDS-UPDRS Part III scores during ON states, suggesting a reversal of motor symptoms [36]. These encouraging findings support the feasibility of early DMT strategies targeting α-synuclein for PD patients.

Small molecule inhibitors targeting α-synuclein are also under development. By directly binding to α-synuclein, they prevent its aggregation or promote its disaggregation, thereby reducing toxic accumulation within neurons. Minzasolmin (UCB0599) is an oral, blood-brain barrier (BBB)-permeable small molecule inhibitor of α-synuclein misfolding. In mouse models, Minzasolmin has shown efficacy in reducing α-synuclein pathology, improving gait and balance, and normalizing dopamine transporter levels in the striatum [37]. Phase I clinical studies of Minzasolmin (NCT04875962) showed acceptable safety and tolerability [38]. Phase Ⅱ clinical trials, namely the ORCHESTRA study (NCT04658186) enrolled 450 early-stage PD patients to assess the efficacy and safety of Minzasolmin, with MDS-UPDRS total scores of Part I - Ⅲ as the primary endpoint. However, on December 16, 2024, the research institution announced the termination of the study's extension phase due to a lack of clinical benefit in the primary endpoints (www.ucb.com/newsroom/press-releases/articlefindings-from-minzasolmin-proof-of-concept-orchestra-study-shape-next-steps-in-ucb-parkinson-s-research-program). Although Minzasolmin is designed to treat PD by preventing the misfolding of α-synuclein, its inhibitory effect on α-synuclein misfolding may be insufficient in practical applications.

**Table 1:** Clinical Trials of DMT for PD.

Targets or strategies	Classification	Drugs	Recruitment criteria	Primary outcomes	Status
**α-Synuclein**					
	Monoclonal antibody	Cinpanemab (BIIB054)	357 early-stage PD patients	MDS-UPDRS total score at weeks 52 and 72	Completed phase 2 trial (NCT03318523)
		Prasinezumab (PRX002)	316 early-stage PD patients	MDS-UPDRS total score at weeks 52	Ongoing phase 2 trial (NCT03100149)
	Small molecule inhibitors	Minzasolmin (UCB0599)	450 early-stage PD patients	MDS-UPDRS total score from baseline up to 18 months	Completed phase 2a trial (NCT04658186)
		Anle138b	68 healthy volunteers	Safety and tolerability	Completed phase 1 trial (NCT04208152)
			70 mild to moderate PD patients	Safety and tolerability in the fasted or fed state	Completed phase 1 trial (NCT04685265)
	Vaccines	PD01A	24 PD patients (Hoehn and Yahr Stage 1-2)	Safety and tolerability	Completed phase 1 trial EudraCT (2011-002650 -31)
		PD03A	36 early-stage idiopathic PD patients	Safety and tolerability	Completed phase 1 trial EudraCT (2014-000568-16)
***LRRK2***					
	LRRK2 inhibitor	BIIB122 (DNL151)	186 healthy volunteers	Safety and tolerability	Completed phase 1 trial (NCT04557800)
			36 PD patients	Safety and tolerability	Completed phase 1b trial (NCT04056689)
			8 healthy volunteers	Absolute bioavailability	Completed phase 1 trial (NCT05005338)
			7 healthy male volunteers	Absorption, metabolism, and excretion	Completed phase 1 trial (NCT05119790)
			84 healthy adult Japanese, Chinese, and Caucasian volunteers	Pharmacokinetics	Completed phase 1 trial (NCT05229562)
			640 early-stage PD patients	Time to Confirmed Worsening in MDS-UPDRS Parts II and III	Ongoing phase 2b trial (NCT05348785)
			400 early-stage PD patients with specific *LRRK2* genetic variants	Time to Confirmed Worsening in MDS-UPDRS Parts II and III	Terminated phase 3 trial (NCT05418673)
		DNL201 (GNE-7915)	29 PD patients	Safety and tolerability	Completed phase 1b trial (NCT03710707)
	*LRRK2* ASO	BIIB094	82 PD patients (≤7 years after diagnosis)	Safety and tolerability	Completed phase 1 trial (NCT03976349)
	LRRK2 PROTAC	ARV-102	Healthy volunteers	Safety and tolerability	Ongoing phase 1 trial
***GBA1***					
	GCase chaperone	Ambroxol	23 PD patients with and without *GBA1* mutations	Level of ambroxol in CSF	Completed phase 2 trial (NCT02941822)
			55 PD dementia patients	Cognitive functions from baseline to week 26 and 52	Ongoing phase 2 trial (NCT02914366)
			65 *GBA1*-PD patients	Cognitive functions from baseline to week 52	Ongoing phase 2 trial (NCT05287503)
			80 *GBA1*-PD patients	MDS-UPDRS Parts III score from baseline up to week 60	Ongoing phase 3 trial (NCT05830396)
	GCase allosteric activator	LTI-291 (BIA-28-6156)	40 *GBA1*-PD patients	Safety and tolerability	Completed phase 1b trial Nederlands Trial Register (NTR6960)
			237 PD patients	Time to Confirmed Worsening in MDS-UPDRS Parts II and III	Ongoing phase 2 trial (NCT05819359)
	Glucosylceramide degradant	Venglustat	273 *GBA1*-PD patients	Part 1: Safety and tolerability Part 2: MDS-UPDRS Parts II+III total score from baseline up to 18 months	Terminated phase 2 trial (NCT02906020)
**PINK1-Parkin axis**					
	PINK1 activator	MTK458 (ABBV-1088)	66 Asian healthy volunteers	Safety, tolerability and pharmacokinetics	Ongoing phase 1 trial (NCT06579300)
			48 healthy volunteers	Safety, tolerability and pharmacokinetics	Ongoing phase 1 trial (NCT06414798)
**Mitochondrial autophagy**					
	Biosynthetic precursor of NAD+	Nicotinamide riboside	30 newly diagnosed, treatment-naive PD patients	Parkinson's disease related pattern measured by FDG-PET	Completed phase 1 trial (NCT03816020)
			80 PD patients	Optimal biological dose	Ongoing phase 2 trial NCT05589766
			400 early-stage PD patients	MDS-UPDRS total score at week 52	Ongoing phase 2 trial NCT03568968
**Endoplasmic Reticulum stress**					
	Neurotrophic factor	Cerebral Dopamine Neurotrophic Factor	17 PD patients	Safety, tolerability and catheter implantation accuracy	Completed phase 1 trial (NCT03295786 and NCT03775538)
**Stem Cell-based Therapy**					
	Fetal nigral tissue	NA	34 advanced PD patients	MDS-UPDRS Part Ⅲ scores	Completed phase 2 trial (NCT00004387)
	Human embryonic stem cells	MSK-DA01	12 PD patients	Safety and tolerability	Completed phase 1 trial (NCT04802733)
	induced human pluripotent stem cells	CT1-DAP001	7 PD patients	Acceptability and safety	Ongoing phase 1/2 trial (NCT06482268)
		NA	3 PD patients	Safety and tolerability	Ongoing phase 1 trial (NCT06145711)
**GLP-1R**					
	GLP-1R agonist	Exendin-4	45 moderate PD patients	MDS-UPDRS Part Ⅲ scores in the “OFF” state from baseline up to 12 and 14 months	Completed phase 2 trial (NCT01174810)
		Exenatide	62 moderate PD patients	MDS-UPDRS Part Ⅲ scores in the “OFF” state from baseline up to week 60	Completed phase 2 trial (NCT01971242)
			194 PD patients	MDS-UPDRS Part Ⅲ scores at 96 weeks	Ongoing phase 3 trial (NCT04232969)
		Lixisenatide	156 early-stage PD patients (< 3 years after diagnosis)	MDS-UPDRS Part Ⅲ scores in the “ON” state from baseline up to 12 months	Completed phase 2 trial (NCT03439943)
	GLP-1 agonist	Liraglutide	63 idiopathic PD patients	MDS-UPDRS Part Ⅲ scores, Non-Motor Symptoms Scale and Mattis Dementia Rating Scale in the “OFF” state from baseline up week 54	Completed phase 2 trial (NCT02953665)

Consequently, it fails to effectively block the aggregation of α-synuclein and the formation of pathological structures such as Lewy bodies, thereby being unable to significantly alleviate the symptoms of patients. This study lacks directly translatable biomarkers reflecting α-synuclein pathology and, crucially, did not employ biomarker stratification (e.g., based on *SNCA* mutation status or α-synuclein burden), resulting in dilution of the signal from responsive patients. Furthermore, although PET tracers demonstrate that Minzasolmin penetrates the BBB and achieves widespread brain distribution [39], its concentrations may be insufficient in specific regions (e.g., the substantia nigra) to effectively inhibit α-synuclein aggregation. Consequently, higher doses or extended treatment durations might be necessary to reach therapeutic thresholds in these critical areas. Anle138b is a small molecule that binds to α-synuclein and inhibits oligomer aggregation. In its Phase I clinical trial, treatment with daily doses of 100-300 mg exhibited good safety profiles [40]. Further studies are needed to explore its efficacy in PD.α-Synuclein vaccines are being investigated with the aim of inducing antibody production or T-cell immune responses to clear or block the aggregation of α-synuclein in the brain. PD01A is one of the representative α-synuclein vaccines, and early clinical trials have shown that PD01A can successfully induce the production of antibodies targeting pathological α-synuclein aggregates [41]. However, there is currently no clear clinical evidence showing that this vaccine significantly slows disease progression. PD03A, an improved version of PD01A, aims to enhance the intensity and specificity of the immune response. Phase I clinical trial data indicate that PD03A is both safe and well-tolerated. Compared to placebo, the 15 μg PD03A treatment group showed statistically significant differences in serum antibody levels after the second (P=0.0189) and fourth treatments (P=0.0258). The 75 μg treatment group also showed significant differences from the second (P=0.0175) to the fourth treatment (P=0.0175). This suggests that PD03A can effectively induce an antibody response and maintain antibody levels for an extended period. Nevertheless, the exploratory results did not demonstrate improvements in either motor or non-motor symptoms [42]. A major challenge for vaccine therapy is ensuring that the antibodies generated can effectively cross the blood-brain barrier and target pathological α-synuclein in the brain.

### 3.2 SNCA

α-Synuclein is encoded by SNCA. Targeting SNCA transcription is also one of the strategies for DMTs in PD. These strategies include [43]: ASOs or siRNAs which can recognize SNCA mRNA level; CRISPR/Cas9, which can delete alleles of *SNCA* or edit mutation sites in the gene; small molecule compounds regulate gene expression.

In primate models, a 4-week ASO treatment has been shown to reduce endogenous α-synuclein levels [44]. One challenge with ASOs is their poor BBB permeability, which requires direct injection into the cerebral ventricles to bypass the BBB. Intranasal delivery, however, is a non-invasive method for CNS drug delivery [45]. siRNAs are artificially synthesized non-coding small RNAs that specifically target mRNA to reduce gene expression by triggering endogenous RNA interference. Richter et al. used polyethylenimine nanoparticles to deliver siRNAs, enhancing their delivery efficiency in the CNS. They found that non-toxic doses of siRNA (0.75 μg) could reduce SNCA mRNA by 67% [46]. The CRISPR-Cas9 system uses short guide RNAs to modify mutated gene loci and reverse the effect of pathogenic mutations. In a PD rat model with *SNCA*-A53T overexpression leading to severe motor impairment, the CRISPR-Cas9 system successfully rescued the overexpression of α-synuclein, dopamine neuron degeneration, and motor symptoms caused by the A53T mutation [47]. Among these strategies, BBB permeability remains a significant challenge. One promising approach is the identification of small molecules that interfere with RNA transcription. Posiphen, for example, has been shown to reduce α-synuclein levels in the brains of mice following 21 days of treatment with 50 and 65 mg/kg doses [48]. However, these studies are still limited to preclinical research, and their efficacy and safety require further validation in clinical trials. While gene therapy represents an exciting avenue for PD treatment, overcoming challenges such as BBB penetration, delivery efficiency, and long-term effects remain crucial for advancing these strategies into clinical applications.

### 3.3 LRRK2

*LRRK2* is one of the most common pathogenic genes for PD [49]. Several LRRK2 mutations associated with PD have been identified, including N1437H, R1441G/C/H, Y1699C, G2019S, and I2020T [50]. LRRK2 is recruited to the membrane by Rab GTPases, where it dimerizes or tetramerizes, and its kinase domain becomes phosphorylated and activated [51, 52]. The G2019S mutation, the most common mutation of LRRK2, which is found in about 1% of sporadic PD cases and 4% of familial PD patients [53], leads to a 2-3-fold increase in LRRK2 kinase activity [54]. Overactivation of LRRK2 causes lysosomal dysfunction [55], exacerbating neuronal degeneration. After activation, LRRK2 can also phosphorylate Rab proteins, such as Rab8 and Rab10, which are involved in vesicular transport. Phosphorylation of these proteins leads to the accumulation of dysfunctional Rabs, thereby impairing vesicular transport [56]. Additionally, the S71R mutation in Rab32, which is autosomal dominant, increases LRRK2 kinase activity [57]. Interestingly, even in other sporadic PD patients who do not carry LRRK2 pathogenic mutations, LRRK2 kinase activity in dopamine neurons is significantly higher than in healthy individuals [55]. This suggests that targeting LRRK2 kinase activity could have therapeutic benefits for more PD patients. Currently, around 40 LRRK2-targeted therapies are in development globally, with several small-molecule drugs designed based on structural targeting already entering clinical Phase Ⅱ/Ⅲ trials [50, 58].

Among these, the most advanced is the LRRK2 inhibitor BIIB122 (DNL151), jointly developed by Biogen and Denali Therapeutics. Seven clinical trials have been conducted to evaluate its potential in PD intervention [50]. The Phase Ⅲ LIGHTHOUSE study, targeting PD patients with LRRK2 mutations, began in September 2022, though it is expected to be completed in 2031 [58]. In June 2023, due to the complexity of the study and its extended duration, Biogen adjusted the research, allocating participants to the Phase Ⅱb LUMA study in order to obtain earlier clinical data on the effects of BIIB122 in PD patients with or without LRRK2 mutations (https://investors.biogen.com/news-releases/news-release-details/statement-biogen-provides-update-parkinsons-disease-clinical).

Another small-molecule compound, DNL201 (GNE-7915), is being evaluated in a Phase Ib randomized controlled trial. In this trial, 29 PD patients were randomly assigned to low-dose, high-dose DNL201, or placebo groups. After 28 days of treatment, DNL201 reduced serum LRRK2 phosphorylation levels by over 50% (https://denalitherapeutics.gcs-web.com/node/7361/pdf).

Although repeated administration of DNL201 in non-human primate models resulted in minor pulmonary morphological changes [59], which raised concerns about the safety of LRRK2-targeted therapies, subsequent research indicated that these changes did not lead to organ dysfunction and could be reversed after discontinuation of the drug [60]. A genetic study on LRRK2 loss-of-function mutations indicated that reduced expression of LRRK2 is not associated with any specific phenotype or disease state [61]. Therefore, excessive concern about the side effects of LRRK2-targeted therapeutic strategies is unnecessary.

Similar to *SNCA*, *LRRK2* ASOs have also emerged as promising treatment strategies. A Phase I clinical trial of BIIB094, an LRRK2 ASO, started in August 2019 and is ongoing until August 2024, with no results currently disclosed (NCT03976349).

In recent years, strategies using PROTACs to induce the degradation of LRRK2 have also attracted significant attention. PROTACs can link E3 ligases to LRRK2, facilitating its degradation through the ubiquitin-proteasome system. Since the first report of PROTACs in 2001 [62], several PROTACs have entered clinical research. One such PROTAC, ARV-102 developed by Arvinas, which acts as an LRRK2 degrader. In non-primate animal models, oral administration of 5 mg/kg ARV-102 resulted in a nearly 90% reduction in LRRK2 levels. Arvinas announced the preliminary results of the Phase I trial of ARV-102 at the 2025 International Conference on Alzheimer's and Parkinson's Diseases (AD/PD™2025). This is a randomized, double-blind, controlled trial, which is divided into a single ascending dose (SAD) cohort and a multiple ascending dose (MAD) cohort. When the single-dose of ARV-102 is ≥ 60 mg or the multiple-dose is ≥ 20 mg, a reduction of more than 90% in peripheral LRRK2 and more than 50% in LRRK2 in the CSF has been observed (https://ir.arvinas.com/news-releases/news-release-details/arvinas-presents-first-human-data-investigational-oral-protac).

### 3.4 GBA1

*GBA1* gene encodes the glucocerebrosidase (GCase), a lysosomal enzyme essential for maintaining lysosomal homeostasis. Approximately 5-10% of PD patients carry at least one *GBA1* mutation [63]. PD patients with severe *GBA1* mutations tend to develop the disease earlier and are more likely to experience cognitive decline [64, 65] and other non-motor symptoms [66]. Mutations in *GBA1* lead to GCase dysfunction [67, 68], and reduced GCase activity results in the accumulation of substrates in lysosomes, leading to lysosomal dysfunction, which accelerates pathological α-synuclein aggregation and neuronal damage [67-70].

Ambroxol, a commonly used expectorant, has recently been found to enhance GCase activity and improve lysosomal function [71]. In early clinical trials, Ambroxol has shown some efficacy. A Phase Ⅱ clinical trial in 2020 involved 17 PD patients who received a maximum dose of 1.26 g of Ambroxol daily for 186 days. The results indicated that Ambroxol was able to effectively penetrate the BBB and, by modulating GCase activity, reduced the MDS-UPDRS Part Ⅲ motor scores by 6.8 points [72]. Although this study lacked a placebo group, the drug showed good safety and tolerability. However, the therapeutic efficacy of Ambroxol still requires confirmation through further clinical studies. A Phase Ⅱ clinical trial initiated in 2015 (NCT02914366) has been investigating the efficacy of Ambroxol at a maximum dose of 1050 mg/day for the treatment of Parkinson's disease dementia (PDD). Similarly, a Phase Ⅱ clinical trial initiated in 2022 (NCT05287503) is evaluating Ambroxol at a maximum dose of 1200 mg/day as a DMT for GBA1-associated Parkinson's disease (GBA1-PD). Both studies focus on the effects of Ambroxol on cognitive function in PD patients. Furthermore, a Phase Ⅲ clinical trial launched in 2023 (the GREAT study, NCT05830396) is assessing Ambroxol at a maximum dose of 1800 mg/day for early-stage GBA1-PD, aiming to determine its efficacy in improving motor function. All three studies are currently ongoing.

The GCase allosteric activator LTI-291 (BIA-28-6156) was investigated in a Phase Ⅰb clinical trial that enrolled 40 GBA1-PD patients. The participants received 10, 30, or 60 mg of LTI-291 or a placebo for 28 days. The results showed that LTI-291 was well-tolerated and reached concentrations in plasma and cerebrospinal fluid sufficient to double GCase activity [73]. Its efficacy is currently being evaluated (NCT05819359).

Venglustat, a drug that targets the degradation substrate of GCase, glucosylceramide, has been tested in the treatment of *GBA1* mutation-related PD. In the Part 1 of the Phase Ⅱ clinical trials (MOVES-PD trial, NCT02906020), Venglustat was found to be safe and well-tolerated in healthy volunteers [74]. In Part 2, Venglustat significantly reduced glucosylceramide levels in plasma and cerebrospinal fluid. However, despite the biochemical improvements, the clinical symptoms of patients worsened, and the risk of psychiatric disorders increased [75]. The failure of Venglustat indicates that inhibiting GCase may not be an effective treatment strategy, or the role of this mechanism in PD has been overestimated. In this study, although the concentration of GCase decreased significantly, other biomarkers such as neurofilament light chain (NfL) increased, and the density of the dopamine transporter decreased. These changes may reflect the acceleration of neurodegeneration, and the disease mechanisms involved require more in-depth research. Furthermore, patients carrying milder mutations (N370S) exhibited a greater worsening in MDS-UPDRS scores following Venglustat treatment (with an inter-group difference reaching 8.0 points), while those with severe mutations (L444P) showed no significant difference. This divergent response may relate to the varying impact of the mutations on enzyme activity. Milder mutations likely retain partial enzyme function; consequently, drug-induced inhibition of gluco-sylceramide synthesis may paradoxically disrupt the compensatory mechanisms of the residual enzyme, leading to metabolic imbalance. Patients with the N370S mutation may possess a more complex lipid metabolic network, rendering single-target intervention insufficient to reverse the pathological cascade. It is important to note that the trial primarily relied on the MDS-UPDRS to assess motor symptoms, a scale known for its limited sensitivity in detecting early disease-modifying effects. Moreover, the assessment of non-motor symptoms (e.g., cognitive function) might not have adequately captured potential neuroprotective effects of the drug. The failure of Venglustat highlights the critical importance of precise patient stratification and rational design of research in future PD clinical trials.

Additionally, several animal model studies have shown that *GBA1* gene therapy, using AAV vectors, can increase GCase maturation and its activity, reduce pathological α-synuclein aggregation, and rescue cognitive and fine motor functions in mice [76]. Currently, several AAV gene therapies have been approved for clinical use, with indications including hemophilia, Duchenne muscular dystrophy (DMD), and spinal muscular atrophy (SMA). However, due to the poor BBB permeability of AAV vectors, their application in the central nervous system remains limited. Viviana Gradinaru et al. developed various AAV vectors, including AAV-MaCPNS and AAV-MaCPNS2, which are capable of crossing the BBB [77]. The CNS expression efficiency of AAV-PHP.B is at least 40 times higher than that of the standard vector [78]. Recently, a new AAV vector has been developed that can bind to the human transferrin receptor, which is highly expressed in the BBB. When the AAV-GBA1 vector is injected into mice expressing the human transferrin receptor, the vector is able to cross the BBB and be delivered to most cells throughout the brain [79].

### 3.5 PINK1-Parkin axis

In 1998 [80] and 2001 [81], the genetic factors for early-onset PD were identified as *PRKN* and *PINK1*, respectively. *PINK1* encodes PINK1 [82], a highly conserved kinase expressed in the mitochondria. *PRKN* encodes E3 ubiquitin-protein ligase Parkin [83], which is a substrate of PINK1. When mitochondria are damaged, the mitochondrial membrane potential depolarizes, activating PINK1, which subsequently phosphorylates ubiquitin and Parkin [84]. Subsequently, Parkin translocates from the cytoplasm to the damaged mitochondria, where it acts as a marker and initiates mitophagy [85]. This process plays a crucial role in mitochondrial function and quality control. When *PRKN* and *PINK1* mutations associated with PD are present, the loss of function of Parkin and PINK1 reduces mitochondrial autophagy, leading to the accumulation of damaged mitochondria and neuronal death [86].

Currently, the development of PD treatments targeting the PINK1-Parkin axis is still in the early stages. MTK458 (ABBV-1088), developed by Mitokinin, is an oral, BBB-penetrating small molecule compound that binds to PINK1 and stabilizes its activity, thereby increasing mitochondrial autophagy [87]. In October 2023, AbbVie acquired Mitokinin and continued to invest in the research process. Two Phase I clinical trials (NCT06579300 and NCT06414798) have been registered and are currently recruiting participants. The plant extract celastrol has been shown in mouse models to enhance mitochondrial autophagy, reverse the damage caused by 1-methyl-4-phenyl-1,2,3,6-tetrahydropyridine hydro-chloride (MPTP) to dopaminergic neurons, and restore motor function [88].

### 3.6 Neuroinflammation and GLP-1R

There is a significant interplay between diabetes and PD. Epidemiological studies indicate that individuals with diabetes exhibit a 38% increased risk of developing PD [89]. Early research has demonstrated that the incidence of PD is reduced in patients with Type 2 diabetes mellitus (T2DM) who are treated with anti-diabetic medications [90]. A cohort study involving 89,073 individuals with T2DM found that the incidence of PD in patients using glucagon-like peptide-1 receptor agonists (GLP-1RAs) was 23% lower than in those using dipeptidyl peptidase 4 inhibitors (DPP4i) [91]. These findings have fueled increased research interest in obesity drugs, a blockbuster class of medications, particularly their potential neuroprotective effects on PD. Many studies now hypothesize that the underlying mechanisms may involve the suppression of neuroinflammation. Neuro-inflammation is a hallmark of PD, which exacerbates disease progression through interactions with PD-associated gene products, mitochondrial dysfunction, and oxidative stress [92]. In the brains of PD patients, elevated levels of inflammatory cytokines in neurons and activation of microglial cells suggest a neuroinflammatory environment [93, 94]. This neuroinflammation amplifies pre-existing harmful genetic factors, contributing to the degeneration of dopaminergic neurons in PD [93, 95]. Long-term use of nonsteroidal anti-inflammatory drugs (NSAIDs), such as ibuprofen, has been shown to reduce the incidence of PD by 46% compared to age-matched non-users [96], thus confirming the close relationship between neuroinflammation and PD. GLP-1RAs, widely used in the treatment of T2DM, exert their anti-inflammatory effects by inhibiting microglial activation and reducing the expression of tumor necrosis factor α (TNF-α), interleukin-1β (IL-1β), and other proinflammatory molecules, thereby protecting dopaminergic neurons [97].

Several GLP-1RAs are currently available, with Exendin-4 being the first to be utilized in clinical studies related to PD (NCT01174810) [98]. This study enrolled 45 moderate PD patients who were randomly assigned to either the treatment or control group. In the treatment group (n=21), patients received subcutaneous injections of 5 mg Exendin-4 twice daily for the first month. From the second to the twelfth month, the dose was increased to 10 mg, followed by a two-month wash-out period. At the 12-month mark, the MDS-UPDRS Part Ⅲ score in the “OFF” state (following overnight discontinuation of conventional PD medication) revealed a 2.7-point improvement in the treatment group, while the control group worsened by 2.2 points (P=0.037). These results suggest a potential disease-modifying effect of Exendin-4 on PD, with benefits persisting for up to 12 months post-treatment cessation. Due to the lack of commercial sponsorship, the study was designed as a single-blind trial, and the results should be interpreted with caution. Subsequently, the same research team conducted a randomized, double-blind, placebo-controlled study (EXENATIDE-PD study, NCT01971242) involving 62 moderate PD patients. Participants received 2 mg Exenatide (a synthetic form of Exendin-4) or placebo via weekly subcutaneous injection for 48 weeks, followed by a 12-week wash-out period. At week 60, the MDS-UPDRS Part Ⅲ score after an overnight drug withdrawal indicated a 1.0-point improvement in the treatment group, while the placebo group worsened by 2.1 points (P=0.0318) [99]. Recruitment for the Phase Ⅲ clinical trial (Exenatide-PD3 study, NCT04232969) has been completed, but results have not yet been reported.

Lixisenatide, another GLP-1RA, was evaluated in a Phase Ⅱ clinical study involving 156 early-stage PD patients (less than 3 years post-diagnosis). Patients were administered 20 μg of lixisenatide or placebo via daily subcutaneous injection for 12 months, followed by a 2-month wash-out period. At 12 months, the MDS-UPDRS Part Ⅲ score in the “ON” state showed a 3.08-point improvement in the treatment group compared to placebo (P=0.007) [100].

In a Phase Ⅱ trial of liraglutide, a GLP-1 agonist, 63 PD patients were randomized to receive 1.2 mg or 1.8 mg of liraglutide, or placebo, once daily for 52 weeks. At week 54, the Non-Motor Symptoms Scale (NMSS) score in the “OFF” state indicated a 6.6-point improvement in the treatment group, whereas the control group worsened by 6.5 points (P=0.07), with no significant differences observed in the MDS-UPDRS Part III score [101].

PD is caused by the interaction of environmental and genetic factors, with certain gene mutations increasing the body's sensitivity to environmental factors [102]. Although studies on the concordance rate of PD have shown a rate of only 27%, suggesting that genetic factors play a lesser role in the pathogenesis of PD compared to environmental factors, research on specific genes and targeted therapies remains a key focus in the study of PD treatment. A thorough understanding, and even the reversal, of the neuronal damage caused by gene mutations is expected to be the future direction for PD patients, at least for certain specific patient groups.

### 3.7 Cellular Organelle Function Repair Therapy

In PD, mitochondrial dysfunction leads to insufficient energy metabolism and elevated reactive oxygen species (ROS), which damages dopaminergic neurons. Additionally, the activity of mitochondrial complex I is significantly reduced, leading to decreased efficiency in the mitochondrial respiratory chain, thereby increasing oxidative stress. This phenomenon is particularly evident in dopaminergic neurons in the SNc [103]. PD-related gene mutations, such as *PINK1*, *PRKN*, and *DJ-1*, cause defects in mitochondrial autophagy and mitochondrial quality control, ultimately resulting in neuronal death [104]. Targeting mitochondrial dysfunction with therapeutic strategies could provide effective interventions for PD.

Nicotinamide riboside, a precursor to Nicotinamide adenine dinucleotide (NAD+), is an important cofactor for mitochondrial respiration, and its reduced form, NADH, plays a key role in cellular energy metabolism. Supplementing NAD+ can help repair mitochondrial dysfunction [105]. In the NADPARK study (NCT03816020, Phase Ⅰ), 30 newly diagnosed PD patients received 1000 mg of nicotinamide riboside for 30 days. The drug was able to penetrate the brain and significantly increased NAD+ levels in the brains of PD patients. A trend toward a reduction in MDS-UPDRS scores was observed in the subgroup with elevated NAD+ [105], but the results should be interpreted with caution due to the limited sample size and observation period. Currently, Phase Ⅱ clinical trials of nicotinamide riboside are ongoing (NCT05589766, NCT03568968).

Lysosomes are vesicles within cells that contain acidic hydrolases, primarily responsible for protein degradation. Imbalance in lysosomal function has been associated with various neurodegenerative diseases [106, 107]. Genetic data from multiple studies indicate that mutations in lysosome-associated proteins are linked to PD [108, 109]. Several genetic risk factors for PD are also related to lysosomal dysfunction, such as mutations in *GBA1*, *LRRK2*, *SMPD1* (*Sphingomyelin Phosphodiesterase 1*), *TMEM175* (*Transmembrane Protein 175*), *SCARB2* (*Scavenger Receptor Class B Member 2*), and others [110]. Additionally, interactions between PD-related genes (e.g., *Rab7L1* and *LRRK2*) can lead to lysosomal dysfunction [111]. When lysosomal function is impaired, undigested metabolites accumulate within lysosomes, resulting in lysosomal storage disorders (LSDs). The metabolites accumulated in LSDs can specifically interact with and induce α-synuclein aggregation [112].

Rapamycin and its analogs, including CCI-779 and Temsirolimus, enhance autophagy-lysosomal pathway function by inhibiting the mTOR signaling pathway, which can reduce the accumulation of α-synuclein aggregates. These compounds have shown neuroprotective effects in preclinical studies, but their immunosuppressive side effects may limit their clinical application in PD patients [113].

The acidic environment inside the lysosome is crucial for its degradation function. TMEM175, a lysosomal membrane protein that acts as an ion channel, when defective, leads to excessive lysosomal acidification, impaired proteolytic activity, and promotes α-synuclein aggregation. TMEM175 dysfunction has been identified as a PD risk factor [114]. Acute inhibition of TMEM175 by compounds such as 2-phenylpyridin-4-ylamine (2-PPA) and AP-6 can enhance the lysosomal degradation capacity of large molecules [115], making them potential candidates for PD treatment.

Furthermore, recent studies have explored the use of proton pump inhibitors or drugs that regulate lysosomal pH to improve the acidic environment of lysosomes, thereby enhancing its degradation capacity. Animal experiments have shown that these acidification-regulating drugs can effectively improve cellular clearance of α-synuclein by restoring lysosomal acidification [116]. However, most of these therapies are still in the early stages of animal testing and have not yet entered large-scale clinical trials.

Misfolded α-synuclein accumulates in dopaminergic neurons, leading to endoplasmic reticulum (ER) stress and the formation of Lewy bodies. During this process, the ER chaperone protein BiP dissociates from the ER transmembrane proteins inositol-requiring enzyme 1α (IRE1α) and protein kinase R-like ER kinase (PERK). Misfolded proteins directly interact with and activate the transcription factor 6 (ATF6), triggering the unfolded protein response (UPR) [117, 118]. Chronic ER stress and UPR activation are early triggers in PD pathology, accelerating dopaminergic neuronal death [119]. In animal models, the PERK inhibitor GSK2606414 protects dopaminergic neurons and restores motor function by increasing dopamine and synaptic protein levels [120, 121], though concerns about pancreatic toxicity remain [122].

Cerebral Dopamine Neurotrophic Factor (CDNF) regulates the function of IRE1α and PERK. In Phase I clinical trials, CDNF administered via an implanted intracerebral injection catheter showed good safety and increased dopamine transporter (DAT) availability in the striatum of patients, slowing disease progression [123]. However, this intracerebral injection method poses additional risks and surgical complications, leading to concerns and reluctance among some patients.

Various organelles are involved in the pathogenic mechanisms of PD, and their interactions are complex [124-126]. A deeper understanding of the roles and relative importance of different organelles in the development of PD will help uncover strategies to overcome neuronal death at the cellular level.

### 3.8 Stem Cell-based Therapy

The DMT strategies discussed above, whether targeting proteins, genes, or organelles, aim to modify the underlying mechanisms of disease progression in patients. Stem cell-based therapy for PD, as a form of regenerative medicine, focuses on replenishing or replacing the lost dopaminergic neurons in PD patients to restore dopamine levels and alleviate symptoms. Unlike other neurodegenerative diseases, where neuronal loss is widespread, PD specifically involves the loss of dopaminergic neurons in the SNc of the midbrain. Thus, cell replacement therapies might be effective even with the transplantation of a single type of cell.

Cell replacement therapy for PD was first explored clinically as early as 1989 [127]. In a two-year randomized controlled clinical trial, 34 advanced PD patients were randomly assigned to receive fetal nigral tissue transplants from either one or four donors or a sham surgery, with a one-week interval between surgeries. Patients began receiving cyclosporine (6 mg/kg/day) two weeks before the first surgery, which was reduced to 2 mg/kg/day two weeks after the second surgery, and maintained for six months. The primary endpoint observed was the change in the MDS-UPDRS Part Ⅲ scores. The results showed that, compared to the sham surgery, fetal dopaminergic neuron transplants led to long-term survival of the grafted cells. However, there was no significant improvement in patient symptoms in both groups. In the sham group and one-donor group, MDS-UPDRS Part Ⅲ scores worsened by 9.4±4.25 and 3.5±4.23 points, ; and in the four-donor group, it worsened by 0.72±4.05 points (P=0.096 when compared to the sham group). The therapy appeared to be more effective in younger patients or those who had a stronger response to levodopa before surgery [128]. This result was likely influenced by the cessation of immunosuppressive therapy six months post-surgery.

Fetal tissue research faced significant challenges due to limited availability and poor consistency of tissue cells, leading to a stagnation in the field. This situation changed in 1998 with the discovery of human embryonic stem cells (hESCs) [129], which offered improvements in accessibility, standardized preparation, ease of preservation, high purity, and unlimited numbers of transplantable cells [130]. Subsequently, the discovery of human induced pluripotent stem cells (hiPSCs) in 2007 [131] further accelerated research in this area. iPSCs technology is a recent breakthrough, which allows for the reprogramming of adult cells into pluripotent stem cells, similar to hESCs, and can be directed to differentiate into dopaminergic neurons. In August 2023, BlueRock Therapeutics published results from their trial using hESCs. The trial involved low-dose (900,000 cells per putamen, n=5) and high-dose (2.7 million cells per putamen, n=7) cohorts. All 12 patients in both cohorts showed good tolerance to the therapy. Clinical symptoms improved overall, with the low-dose group showing a 7.6-point decrease in MDS-UPDRS Part Ⅲ scores, and the high-dose group showing a 13-point decrease, indicating greater improvement (NCT04802733, www.bluerocktx.com/bluerocks-phase-i-study-with-bemdaneprocel-in-patients-with-parkinsons-disease-meets-primary-endpoint).

iPSCs are receiving much attention due to their ability to reduce the immune response to grafts [132] and avoid the ethical concerns surrounding hESCs. The first clinical case using iPSCs was published in 2020. The patient received two autologous iPSC transplant surgeries, one in the left and one in the right putamen, six months apart, with 4 million cells transplanted each time. Post-surgery, the grafts survived, and the patient did not develop new motor dysfunction. There was no need for immunosuppressive drugs, and clinical benefits were gradually observed between 18 to 24 months post-surgery, including a 6% reduction in levodopa equivalent daily dose, a decrease in MDS-UPDRS Part Ⅲ scores, and an improvement in quality of life [132]. Currently, more clinical trials (NCT06482268, NCT06145711) are underway.

Early concerns regarding stem cell-based therapy for PD included the potential tumorigenicity of grafts, such as the formation of teratomas [133]. A sequencing study on hiPSCs revealed that although mutations are common in hiPSCs, once differentiated into neurons, they do not carry cancer-related gene mutations [134]. Moreover, no tumor formation has been observed in multiple therapeutic studies [135, 136]. Currently, the primary concern lies in the efficacy of stem cell-based therapy. Brain-wide mapping of the monosynaptic inputs of dopaminergic neurons demonstrates that dopaminergic neurons in the SNc receive highly diverse and complicated innervations from broad brain regions and are functionally subcircuit-specific [137, 138]. Although grafts have shown survival in target regions in current studies, the extent to which they can establish the essential functional neural circuits and whether the integration of novel neural circuits brings side effects remain to be further explored.

## 4. Biomarkers for PD

Specific biomarkers play a vital role in PD clinical trials, serving important functions in disease characterization, subject stratification, efficacy monitoring, and other aspects. For example, serum uric acid is negatively correlated with PD [139]; tau and β-amyloid act as biomarkers for cognitive impairment in PD [140]; and the level of NfL may predict more severe motor [141] and non-motor [142] symptoms.

Achieving superior effects of DMTs critically hinges on effective intervention before significant dopaminergic neuron degeneration occurs. Individuals carrying pathogenic mutations who have not yet developed clinical symptoms or biomarker alterations represent a population with the greatest potential to benefit from future early-intervention clinical trials targeting specific pathogenic mutations. Disease early diagnosis constitutes the essential first step in this process. For early disease diagnosis, the most promising candidate is likely α-synuclein, encompassing total, monomeric, oligomeric, and phosphorylated α-synuclein. Among these, total α-synuclein has shown marked variations across different studies, rendering it seemingly unsuitable as a PD biomarker [143]. Furthermore, investigations utilizing α-synuclein antibodies have not demonstrated progressive changes in total α-synuclein levels [144], thus failing to directly reflect disease progression.

The α-synuclein seed amplification assay (αSyn-SAA) leverages the self-replicating capacity of minute amounts of misfolded oligomers to nucleate further aggregation, enabling the detection of α-synuclein aggregates in CSF at concentrations as low as 0.1 pg/mL [145]. Recent meta-analysis findings indicate that αSyn-SAA in CSF, skin, blood, and extracellular vesicles exhibits high sensitivity (86%) and specificity (92%) [146]. However, αSyn-SAA performance varies among different genetic PD subgroups. Results from a 2023 study demonstrated a progressively lower proportion of αSyn-SAA positivity in GBA-PD than in sporadic PD, and lower still in LRRK2-PD [147, 148]. The recent study in 2025, using PPMI clinical and CSF-αSyn-SAA data, demonstrated that kinetic parameters vary across PD subtypes, including time to reach 50% of maximum fluorescence (T50), time to threshold (TTT), and area under the curve (AUC). Results showed that *LRRK2*-PD exhibited longer T50 and TTT, and a smaller AUC compared with *GBA*-PD and sporadic PD. These findings suggest the potential of αSyn-SAA in genetic stratification [148]. αSyn-SAA, as a detection method that has received widespread attention in recent years, its current value lies in confirming PD diagnosis rather than serving as a diagnostic gold standard. It is also difficult to accurately predict disease progression, and more extensive research and optimization are needed before it is truly applied in clinical practice. In the design of clinical studies, exploring subgroup-specific biomarkers is of particular importance.

In PD patients, radioligand uptake by the dopamine transporter (DAT) is reduced. DAT-SPECT, as a quantifiable diagnostic tool, can be used in disease-modifying therapy trials where the specific binding ratio (SBR) serves to confirm and quantify dopaminergic neuronal damage in enrolled patients, thereby improving the homogeneity of the study population. Despite the poor correlation between imaging changes and longitudinal clinical functional changes [149], along with limited spatial resolution, DAT-SPECT has been utilized, particularly in trials exploring neurotrophic factors or Stem Cell-based Therapy, where its measurements may serve as evidence supporting the restoration of dopaminergic neuron function [16]. As an objective endpoint in clinical research, DAT-SPECT holds unique value in assessing disease progression and treatment responses. However, its interpretation is complicated by its poor correlation with temporal changes in clinical function. Additionally, DAT-SPECT imaging may be affected by acute and chronic confounding effects arising from drug interventions, which imparts it with potentially significant limitations as a biomarker for disease progression in clinical trials [150]. Magnetic resonance imaging (MRI), owing to its relatively lower cost and absence of radiation concerns, is employed for monitoring PD across different stages. MRI techniques such as striatal dopaminergic imaging, metabolic imaging, and free-water/neuromelanin-sensitive imaging in the posterior substantia nigra can monitor progression in early PD. Conversely, anterior nigral free-water imaging, nigral R2* relaxation rate, and metabolic imaging are used to track progression in mid-to-late-stage PD patients [150]. These individual modalities possess varying degrees of limitations. Nevertheless, machine learning-based multi-omics frameworks integrating radiomics, biomarkers, and genetic information represent a powerful strategy within the current rapidly evolving artificial intelligence landscape [151, 152].

## 5. Lessons from Failed Trials

Cinpanemab in the PARK study, Minzasolmin from the ORCHESTRA study, and Venglustat in the MOVES-PD trial have all failed to demonstrate satisfactory efficacy. Potential reasons, as discussed above, highlight critical lessons from these setbacks. First, precise patient stratification is paramount. For the same molecular target, distinct mutation subtypes or disease stages necessitate refined stratification to accurately identify potentially responsive PD subpopulations. Both Cinpanemab and Minzasolmin target α-synuclein for the treatment of PD. However, neither study stratified participants according to *SNCA* mutation status or α-synuclein burden, leading to the potential dilution of the population that might derive benefit. Furthermore, α-synuclein aggregation is not central to the pathological features of patients carrying *LRRK2* gene mutations. Such patients show poor responses to α-synuclein-targeted drugs, and neither the Cinpanemab nor the Minzasolmin studies excluded patients with *LRRK2* gene mutations, thereby exerting a certain impact on the results. Similarly, the MOVES-PD trial did not stratify patients based on mild (N370S) and severe (L444P) *GBA1* mutations, with detailed analyses as described above. Second, longitudinal biomarker monitoring and extended follow-up are essential. The MOVES-PD trial only enrolled 221 cases of *GBA1* mutation-related PD, with a trial duration of 1 year, which may be insufficient to detect disease-modifying effects. *GBA1* mutation-related PD has a longer disease course, and changes in glycosphingolipid metabolism may require a longer period to be reflected in clinical scores. Third, the timing of drug intervention should be reasonably selected. In PD patients, the entry of α-synuclein oligomers into cells may represent an early event. Cinpanemab primarily binds to extracellular α-synuclein oligomers, while Minzasolmin, moreover, targets misfolding at an even earlier stage than this early event. However, both Cinpanemab and Minzasolmin were investigated for use in patients with definitively diagnosed PD, potentially missing the optimal intervention window; the preclinical or prodromal stage might instead be a better choice. Fourth, different biomarkers may yield different results. Cinpanemab is based on binding to α-synuclein aggregates for its therapeutic effect. In the PARK study, the αSyn-SAA assay was used to detect α-synuclein aggregates in some participants, yet we did not observe relevant result analyses. Perhaps the acquisition and processing of CSF samples posed difficulties for researchers. Fifth, the clinical relevance of the selected primary endpoints is insufficient. All these studies selected MDS-UPDRS scores as the primary endpoint. Although this metric is commonly used in PD assessments, MDS-UPDRS exhibits high intra-individual variability in long-term follow-up, which may fail to accurately reflect actual disease progression. Meanwhile, it does not distinguish between symptomatic improvement and disease modification. Future trials should incorporate more sensitive biomarkers, broader endpoints (e.g., cognitive function, quality of life), and prolonged follow-up periods to comprehensively assess long-term effects.

## 6. Conclusion

The basic and clinical research on Parkinson's disease faces several challenges:

1. Complexity of Etiology: PD is caused by the interaction of multiple genetic and environmental factors, and the mechanisms of disease progression are complex. Our understanding of these mechanisms remains relatively limited, which complicates accurate stratification of patients in clinical trials. Combining multiple therapeutic mechanisms, especially targeting multiple interconnected targets, may be more beneficial than focusing solely on the primary pathogenic mechanism of a single disease subtype. However, before enhancing the efficacy by adding a second intervention, it is necessary to demonstrate the effectiveness of a single intervention in patient subgroups.
2. Lack of Reliable Biomarkers: Unlike Alzheimer's disease, PD lacks reliable, highly accurate, and sensitive biomarkers with strong accessibility, which hinders patient selection and ongoing evaluation of therapeutic efficacy in clinical trials. Ideally, future studies should establish more comprehensive biobanks of biological samples (such as blood, CSF, urine, hiPSCs, etc.), thereby enabling retrospective evaluation of post-study responses and validation of newly identified biomarkers.
3. Early Pathophysiological Changes: The neurodegenerative processes in PD occur before the onset of symptoms. It is difficult to identify and screen potential ultra-early-stage patients on a large scale. This period might be the most ideal window for disease-modifying treatments.
4. BBB Challenge: The existence of the BBB limits the application of many therapeutic strategies in PD.

In recent years, there has been significant preclinical and clinical research focused on identifying biomarkers for PD, such as α-synuclein, lysosomal enzymes, tau protein, neurofilament light chain protein, among others [153]. However, challenges remain in improving diagnostic accuracy for early-stage patients, distinguishing pathological α-synuclein, and guiding DMT. In the future, the identification of suitable PD-related biomarkers, the exploration of early diagnostic and monitoring methods, and personalized stratification of patients based on clinical features and genetic factors for tailored DMT may hold the key to overcoming PD progression.
